# Targeting the DNA damage response in hematological malignancies

**DOI:** 10.3389/fonc.2024.1307839

**Published:** 2024-01-29

**Authors:** Sanjay De Mel, Ainsley Ryan Lee, Joelle Hwee Inn Tan, Rachel Zi Yi Tan, Li Mei Poon, Esther Chan, Joanne Lee, Yen Lin Chee, Satish R. Lakshminarasappa, Patrick William Jaynes, Anand D. Jeyasekharan

**Affiliations:** ^1^ Department of Haematology-Oncology, National University Cancer Institute, Singapore, National University Health System, Singapore, Singapore; ^2^ Department of Medicine, Yong Loo Lin School of Medicine, National University of Singapore, Singapore, Singapore; ^3^ NUS Center for Cancer Research (N2CR), Yong Loo Lin School of Medicine, National University Singapore, Singapore, Singapore; ^4^ Cancer Science Institute of Singapore, National University of Singapore, Singapore, Singapore; ^5^ Department of Anatomy, Yong Loo Lin School of Medicine, National University of Singapore, Singapore, Singapore

**Keywords:** DNA damage, inhibitors, combination therapy, haematologic malignancies, clinical trials

## Abstract

Deregulation of the DNA damage response (DDR) plays a critical role in the pathogenesis and progression of many cancers. The dependency of certain cancers on DDR pathways has enabled exploitation of such through synthetically lethal relationships e.g., Poly ADP-Ribose Polymerase (PARP) inhibitors for BRCA deficient ovarian cancers. Though lagging behind that of solid cancers, DDR inhibitors (DDRi) are being clinically developed for haematological cancers. Furthermore, a high proliferative index characterize many such cancers, suggesting a rationale for combinatorial strategies targeting DDR and replicative stress. In this review, we summarize pre-clinical and clinical data on DDR inhibition in haematological malignancies and highlight distinct haematological cancer subtypes with activity of DDR agents as single agents or in combination with chemotherapeutics and targeted agents. We aim to provide a framework to guide the design of future clinical trials involving haematological cancers for this important class of drugs.

## Introduction

DNA damage occurs due to a variety of endogenous or exogenous insults, contributing to genomic instability, a hallmark of cancer (1, 2). DNA damage response (DDR) pathways have therefore evolved to maintain genomic stability, which is essential for safe and stable transfer of genetic information during cell division (1). Cells have distinct, though partially overlapping, pathways dedicated for the repair of distinct forms of DNA damage. These include homologous recombination repair (HR) or non-homologous end joining (NHEJ) (for double strand breaks(DSB)); base excision repair (BER) (for Single strand breaks (SSB), modified bases and abasic sites); nucleotide excision repair (NER) (for UV-induced lesions, and helix distorting adducts), the Fanconi anemia complex (for repairing inter-strand cross links (ICL)) and mismatch repair (MMR) (for mis-incorporated bases, insertions and deletions) (1). For each pathway, a network of DNA damage sensors, transducers and effectors coordinate the identification and repair of DNA in concert with cell cycle progression (3, 4).

**Figure 1:**
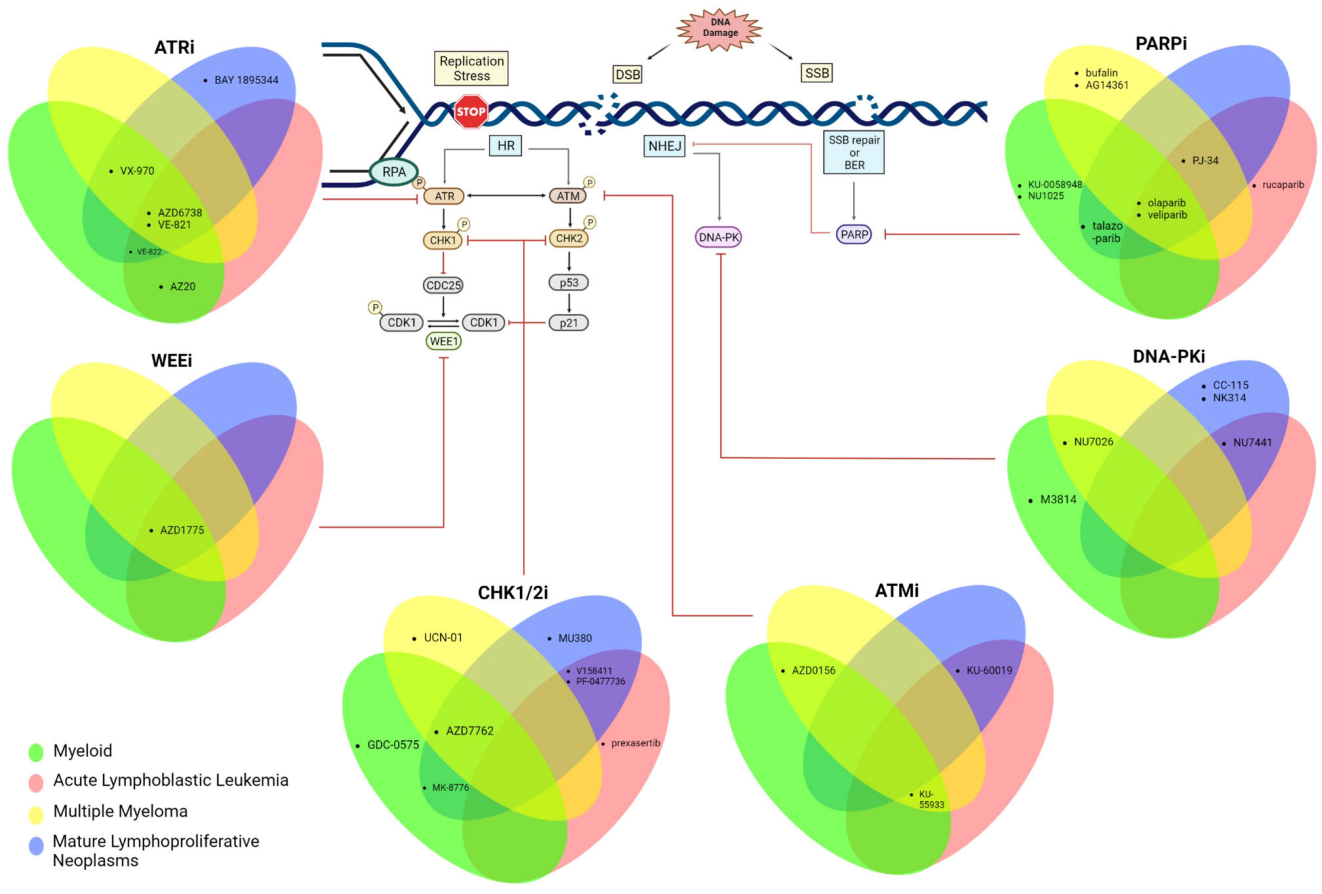
Schematic summarizing the DDR is tested pre-clinically across all haematological malignancies. Created with BioRender.com.

Defects in DDR pathways foster the accumulation of other mutations and are thus an “enabling hallmark” of almost all cancers (2). However, tumors deficient in specific DDR pathways are often over-reliant on other intact DDR pathways for survival, which presents a potential Achilles heel for pharmacologic inhibition via the principle of synthetic lethality (5). A prominent example of this synthetic lethality is the efficacy of Poly (ADP ribose) polymerase 1(PARP1) inhibition in HR-deficient tumors (which are over-reliant on the PARP enzyme for repair of replication associated damage) (6). PARP inhibitors are currently the most advanced DDR inhibitor in terms of clinical development. Although the majority of the clinical data are in the space of epithelial cancer, PARP inhibitors and other DDR inhibitors targeting ATM, ATR, CHK1/2, WEE1 and DNA-PK are being studied in a variety of hematologic neoplasms (7, 8). In this review, we highlight key data supporting DDR inhibition in hematologic malignancies and discuss the opportunities and challenges for the clinical application of these agents. We focus primarily on inhibitors of kinases involved in DSB repair (which constitute the largest group of agents), beginning with the apical kinases and then progress to downstream kinases. For each kinase “node”, we will discuss each malignancy in the following order: myeloid neoplasms, precursor lymphoid neoplasms, multiple myeloma, and mature lymphoproliferative disorders. An overview of the development of DDR inhibitors in haematologic malignancies is shown in **Figure 1**.

## Pre-clinical data for DDR inhibitors in hematological cancers

### Targeting ATM

ATM is a kinase involved in the early signaling of DSBs and is one of the apical sensors of the DSB DDR cascade (9). Its activation results in cell cycle arrest, and impacts multiple downstream pathways including homologous recombination (HR) repair (10). ATM inactivation is a common event in hematological cancers, often coinciding with the loss of a section of the 11q chromosomal arm (11–15). Aberrations at the ATM locus have been observed in Chronic Lymphocytic Leukemia (CLL) (11, 12), T-cell prolymphocytic leukemia (T-PLL) (14, 15) diffuse large B-cell lymphoma (DLBCL) (16), mantle cell lymphoma (MCL) (13, 17) and cutaneous T cell lymphoma (CTCL) (18). In CLL, inactivating mutations in the remaining ATM allele, after 11q deletion, result in a worse prognosis than those with loss of one allele alone through 11q deletion (12). Downregulation of ATM expression has also been reported in *IDH1*-mutant AML patients (19). The high incidence of lymphoid malignancies in those with Ataxia Telangiectasia (20), in conjunction with the frequency of somatic ATM aberrations described previously, is supportive of the notion that ATM acts a tumor suppressor in hematological cancers.

While defects in ATM function are now recognized across these cancers, less is known about scenarios that depend on ATM activity- with potential for synthetic lethal targeting. In addition to its role in repairing DNA damage, ATM also has pro-apoptotic functions (21, 22). This suggests that ATM has multidimensional roles in maintaining genomic stability and targeting this kinase could be a double-edged sword. This may be a reason why ATM inhibitors have not been as successful as many other DDR targeting cancer therapeutics. As such, there are hitherto no registered clinical trials for ATM inhibitors in hematological malignancies. Nevertheless, we summarize below, proof-of-principle data generated in preclinical systems which highlight potential clinical scenarios where ATM targeting can be explored.

#### ATM inhibition in myeloid malignancies

ATM signaling ostensibly activates anti-apoptotic NF-kB signaling in an acute myeloid leukemia (AML) cell line, and treatment with the ATM inhibitor KU-55933 increased apoptosis (23). In support of this, CD34+ bone marrow mononuclear cells of high-risk AML patients also undergo apoptosis in response to ATM inhibition, with a concomitant decrease in NF-kB signaling (23). Along a similar vein, the ATM inhibitor, AZD0156 (AstraZeneca), synergized with an inhibitor of anti-apoptotic protein, BFL-1, to induce apoptosis of AML cells (24). AZD0156 treatment resulted in prolonged survival in a murine model of MLL-rearranged AML, a form of AML that is resistant to genotoxic therapy due to an ineffectual p53 response (25). The MLL-AF9 fusion gene leads to leukemogenesis through differentiation arrest (26). Interestingly, perturbation of ATM resulted in terminal differentiation of murine myeloid blasts expressing MLL-AF9 (26), providing rationale for the use of ATM inhibitors for treatment of MLL-rearranged AML. ATM signaling can also influence survival through interaction with other signaling pathways, for example, maintaining antioxidant capacity of the AML blasts via G6PD, resulting in FLT3 inhibitor resistance (27). Indeed, in FLT-3 ITD mutant AML, which possess FLT-3 activating mutations, the FLT-3 inhibitor, Quizartinib, was synthetically lethal with ATM loss (27). These data collectively highlight the potential for targeting ATM in AML, with the MLL rearranged subset being the most likely to benefit. The question of whether efficacy may be enhanced by combining ATM inhibitors with other targeted agents remains to be answered.

#### ATM inhibition in acute lymphoblastic leukemia

As with AML, ATM signaling to the NF-kB pathway promotes tumor survival in acute lymphoblastic leukemia (ALL). In ALL cell line models, chemotherapy upregulated chemoprotective cytokines in an ATM and NF-kB dependent manner (28). Congruent with this, inhibition of either ATM using KU-60019 or the NF-kB pathway using pyrrolidinedithiocarbamate ammonium (PDTC) enhanced chemo sensitivity in an ALL xenograft model (28). Similarly, inhibition of ATM using KU-55933 increased etoposide-induced apoptosis in Jurkat (T-lymphoblastic leukemia) cell lines, while sparing resting T-cells (29). Further studies are required to evaluate ATM inhibitors in MLL rearranged ALL, as with AML, the selection of optimal combinatorial partners is an important area for future investigation.

#### ATM inhibition in multiple myeloma and mature lymphoproliferative neoplasms

ATM inhibition through KU-55933 has been shown to have limited efficacy against multiple myeloma (MM) cells lines either alone (30, 31) or in combination with doxorubicin (31). However, MEDI2228, a B-cell maturation antigen (BCMA) specific antibody conjugated with the DNA cross-linking agent tesirine, synergized with AZD0156 in MM cells lines (32). MEDI2228 also synergized with Bortezomib, a proteasome inhibitor that perturbs the NFkB pathway and is used as a standard of care for the treatment of MM (32). Synergy with both AZD0156 and Bortezomib is consistent with the observation in AML/ALL cells that ATM signals to the NFkB pathway (see above for AML/ALL). Further studies exploring the interplay between ATM, NF-kB and proteasome function in MM are required. With BCMA targeting immunotherapies becoming a vital part of MM treatment (33), their synergy with ATM inhibition would be of great interest and should be evaluated in future studies.

In Mantle Cell Lymphoma (MCL) cell lines, the combination of the ATM inhibitor KU-60019 with the histone deacetylase (HDAC) inhibitor Romidepsin (34) was found to be synergistic via downregulation of the CDK inhibitor p21 (35). Future studies should evaluate the combination of ATM inhibitors with Bruton Tyrosine Kinase (BTK) and BH3 mimetics in MCL as these combinations are more likely to be taken forward into clinical trials.

### Targeting ATR

ATR is an apical DDR kinase that is critical for initiating cell cycle arrest specifically in the context of replication stress (9). ATR is also postulated to have a direct role in HR repair (36). Unlike with ATM, ATR-initiated signaling is not commonly disrupted in hematologic malignancies (37). In fact, it is often observed to be augmented, for example, in murine BCR-Abl+ myeloid cells and primary chronic myeloid leukemia (CML) cells (37). A subset of MM characterized by chromosomal instability also has an enrichment of genes in the ATR signaling axis (38). In this malignancy, ATR signaling has also been implicated in resistance to the alkylating agent, melphalan, as observed in a resistant MM cell line (39). In contrast to ATM inhibitors, ATR inhibitors are at a more advanced phase of clinical development. The pre-clinical rationale for their use is described below.

#### ATR inhibition in myeloid malignancies

The ATR inhibitor AZ20 has antitumor activity against AML with MLL rearrangements in both *in vitro* models (mouse), and *in vivo* allograft and human xenograft mouse models (25). In MLL-AF9 expressing murine myeloid blasts, perturbation of ATR resulted in terminal differentiation in a similar manner to ATM inhibition (described above) (26), suggesting that like with ATM inhibitors, ATR inhibitors will be effective against this subgroup of AML.

ATR inhibitors synergize with a wide variety of agents in AML. The ATR inhibitors AZ20 and AZD6738 (AstraZeneca) combined with the replication stress-inducing agent, cytarabine, were both shown to be synergistic against AML cell lines and primary patient samples (40). This combination resulted in abrogation of the G2/M checkpoint and increased replication stress (represented by increased γH2AX and increased RPA32-bound chromatin), leading to increased apoptosis (40). In a similar manner, the activity of the ATR inhibitor VE-821 was potentiated by other replication stress inducers, hydroxyurea and gemcitabine, in AML cell lines and primary samples coinciding with abrogation of ribonucleotide reductase (RNR) expression and inhibition of replication fork progression (41). The efficacy of this combination was demonstrated in AML xenografts where VX-970 (an orally bioavailable derivative of VE-821 [Merck]) in combination with gemcitabine induced a significant reduction in graft growth and higher survival rates (41). The combination of VX-970 with hydroxyurea would be an attractive all-oral low-intensity therapeutic option for clinical trials in elderly patients with AML.

RNA polymerase I (Pol I) has been shown to have greater transcriptional activity in AML cells as compared to normal myeloid precursors (42). Inhibition of Pol I promotes G2/M arrest by activating the ATR checkpoint, implying that ATR inhibitors would be appropriate combinatorial candidates (42). Indeed, AZD6738 was shown to be synergistic with Pol I inhibitor, CX5461, in AML cell lines and primary AML samples (42). Finally, the combination of WEE1 inhibition (using AZD1775 (AstraZeneca)) with ATR inhibition (using VE-822) in AML cell lines led to apoptosis via disruption of the G2/M checkpoint and increased replication stress (43). The above data suggest that combining ATR inhibition with replication stress inducers may be an effective strategy for treating AML.

#### ATR inhibition in acute lymphoblastic leukemia

In acute leukemias, VE-822 treatment resulted in reduced output from both RNR and deoxycytidine kinase (dCK) pathways of deoxynucleotide synthesis in ALL cells, with the combination of VE-822 with RNR and dCK inhibition (with 3-AP and DI-82, respectively) being lethal (44). The combination of ATR inhibition and interference with deoxynucleotide triphosphate synthesis is thus a promising strategy for evaluation. In B-ALL cells, as with AML, the pol 1 inhibitor CX5461 activates the ATR pathway and mediates G2 checkpoint arrest, and thus induced apoptosis synergistically in combination with VE-822 (45), providing another alternative combination strategy through exacerbation of replication stress. Treatment of ALL cell lines and primary ALL cells with VE-821 in combination with doxorubicin also proved effective (46).

In an *in vitro* and *in vivo* murine model of infant B-ALL with concomitant activated Ras, AZ20 was synergistic with MEK inhibition (using trametinib), which was corroborated in PDX mouse models (47). The recent discovery that T-ALL with BRCA2 mutations were vulnerable to VE-821 and AZ6738 (48) confirms that reliable biomarkers of sensitivity to ATR inhibition would augment the clinical application of these agents in acute leukemias.

#### ATR inhibition in multiple myeloma

Increased replicative stress leading to dependency on ATR signaling is well described in MM (31, 38), with efficacy of VE-821 and VX-970 demonstrated against MM cell lines and patient samples (31, 38). The alkylator, melphalan, which is frequently used for MM treatment, has shown good synergy with VX-970 *in vitro* and in an *in vivo* orthotopic MM mouse model (31). Combinatorial treatment of VX-970 or VE-821 and ATM inhibitor, KU-55933, demonstrated increased cell death in some MM cell lines (30, 31). However, the greater single agent efficacy seen with ATR inhibitors, compared to ATM inhibitors (see above), suggests that MM is more vulnerable to perturbations in pathways required for managing replication stress. AZD6738 was synergistic with antibody drug conjugate, MEDI2228, in MM cells similar to ATM inhibitor-MEDI2228 combinations (32), suggesting focused exposure to DNA damaging payloads may increase the dependence of MM on both DNA damaging pathways.

#### ATR inhibition in mature lymphoid neoplasms

AZD6738 treatment induced mitotic catastrophe in ATM and p53 defective CLL cells and was synergistic with chemotherapy, these findings being corroborated in xenograft models (49). Synergy between ATR inhibition and defective ATM was also observed in ATM-deficient MCL xenografts treated with AZD6738 (50). AZD6738 was also shown to synergize with CHK1 (AZD7762 [Astra Zeneca]) or WEE1 (AZD1775[Astra Zeneca]) inhibitors against MCL and diffuse large B cell lymphoma (DLBCL) *in vitro* and *in vivo (* 51). In this context, the efficacy of ATR inhibition and synergy of the combinations was independent of ATM, TP53 and MYC mutational/expression status (51). AZD6738 demonstrates efficacy in non-GCB DLBCL cell lines that harbor CDKN2A deletion and high MYC expression (52). A xenograft model using these cell lines also demonstrated efficacy with AZD6738 and that synergy was possible in combination with AZD1775 or Rituximab and Bendamustine (R-Benda) (52).

The ATR inhibitor BAY 1895344 (Bayer) showed efficacy activity against certain lymphoma cell lines and in MCL xenograft models (53). Importantly, the *in vivo* efficacy of this agent appeared greater than that of AZD6738 and the ATR inhibitor, VX-970, suggesting promise for further development (53). In cutaneous T-cell lymphoma cell lines, VE-821 or VE-822 was synergistic with phototherapy, suggesting an potential combination for evaluation in trials (54). Given their high level of replication stress, ATR inhibition is more likely to be successful in aggressive lymphomas with a high proliferative index, and these should be prioritized for future clinical trials.

### Targeting DNA PK

DNA-PK, another apical kinase in the DDR pathway, is a sensor of DSB more prominently in G1 when non-homologous end joining (NHEJ) is in operation (55). DNA-PK is also an important component of the cellular response to replication stress, and is known to be required for activation of CHK1 and CHK2 (56). As with ATR, the activity of this kinase appears to be largely intact across the hematological malignancies. Overexpression of DNA-PK (both at the mRNA and protein level) has been shown to confer an inferior prognosis in CLL patients (57, 58), suggesting this kinase as a potential target for some hematological malignancies. However, there are currently no clinical trials for DNA-PK inhibitors in this context. The pre-clinical evidence underlying the rationale for DNA-PK inhibitors in hematological malignancies is outlined below.

#### DNA-PK inhibition in acute leukemias

The DNA PK inhibitor NU7026 augmented the activity of topoisomerase II poisons against myeloid leukemia cells lines (59). Similarly M3814, a selective DNA-PK inhibitor, potentiated the activity of ionizing radiation as well as the anthracycline topoisomerase II inhibitor, daunorubicin, alone or in combination with cytarabine, in AML cell lines and an AML PDX mouse model; this potentiation was only observed in p53 wild type AML cells (60). M3814 also enhanced the activity of DNA damaging agent, calicheamicin, in AML cells as well as the activity of antibody drug conjugate gemtuzumab ozogomycin in AML xenograft mouse models (61). This combination would be promising for evaluation in clinical trials of relapsed or refractory AML and elderly patients who are not eligible for intensive chemotherapy. DNA PK is also overexpressed in B-ALL and in this context the DNA-PK inhibitor NU7441 potentiated apoptosis in combination with doxorubicin (62), suggesting the potential for a combination trial.

#### DNA-PK inhibition in multiple myeloma

NU7026 showed modest activity against MM cells as a single agent but potentiated the effect of doxorubicin, suggesting that MM cells rely on DNA-PK to repair anthracycline induced DSB (63). NU7026 also potentiated the activity of ionizing radiation on MM cells both as a single agent and in combination with PARP inhibitor, AG14361(Pfizer) (64). Conversely, a separate study suggested that NU7026 improved the survival of MM cell lines treated with radiation (65). These heterogenous results seen with DNA-PK inhibition in MM highlight the need for analysis of DNA-PK inhibitors in a larger breadth of MM models before clinical evaluation.

#### DNA-PK inhibition in mature lymphoid neoplasms

Deletions of the short arms of chromosomes 11 and 17 are makers of treatment refractoriness in CLL (57), contributing to the loss of function of ATM (11, 12) and p53 (66, 67), respectively. Overexpression of DNA-PK was consistently associated with del(11p) and del(17p) and this overexpression (both at the mRNA and protein level) conferred an inferior prognosis in CLL patients (57, 58). NU7441 could sensitize patient cells to fludarabine, chlorambucil (57) and mitoxantrone (58), proffering a strategy to overcome this refractoriness. CC-115 is a dual mammalian target of rapamycin (mTOR) kinase and DNA-PK inhibitor that demonstrated efficacy in CLL patient cells, inducing apoptosis and suppressing cellular proliferation, irrespective of ATM or TP53 mutations (68).

With respect to T-cell lymphomas, the prevalence of DSBs and overreliance on NHEJ in Adult T-cell leukemia lymphoma (ATLL) makes it an attractive target for DNA PK inhibition (69, 70). The dual DNA-PK and topoisomerase II inhibitor, NK314 was active against ATLL *in vitro* and *in vivo (* 71). These data provide the basis for clinical trials of DNA-PK inhibition for ATLL, a malignancy that has limited therapeutic options.

### Targeting CHK1/CHK2

CHK1 and CHK2 are effector proteins downstream of the apical kinases ATR and ATM, transmitting the DNA damage signals that lead to cell cycle arrest, facilitating DNA repair and promoting cell survival (9). CHK1 is a vital kinase in the replication stress response, mediating S phase check point activation, replication fork stabilization and DNA repair in response to ATM signaling (1).Mutations in the CHK kinases are not apparent in hematological malignancies and as with other nodes of the DDR signaling appears largely intact. In fact, increased CHK1 activity is observed in AML patient samples with complex karyotypes (72). Prolonged or increased CHK1 signaling is also seen in CML patient samples and murine myeloid BCR-Abl+ cell lines upon DNA damage, compared to non-transformed samples (37). In T/B ALL patient samples and cells lines, the CHK1 protein itself is overexpressed and constitutively active (73, 74). Importantly, overexpression CHK1 has been associated with poor prognosis in AML and resistance to cytotoxic agents in AML patient samples (75).

The development of CHK1 inhibitors spans more than two decades, however, invariably CHK1 inhibition as a monotherapy has resulted in modest effects and combination with traditional genotoxic agents results in unacceptable toxicities (76). Nevertheless, as described below, numerous biomarker-based or combinatorial strategies have been evaluated preclinically to exploit the anti-cancer activity of CHK inhibitors.

#### CHK1/CHK2 inhibition in myeloid malignancies

AZD7762(AstraZeneca) is an ATP-competitive inhibitor of both CHK1 and CHK2 (77) which induced apoptosis in AML cell lines as well as primary samples. The effect was particularly pronounced in cases with complex cytogenetics as well as FLT-3 ITD mutated cell lines (78, 79). As seen with ATR inhibitors, cytarabine synergized with CHK1 inhibitor MK-8776 (Merck). Incorporation of cytarabine into DNA led to replication associated damage and activation of the ATR/CHK1 pathway, leveraging on the role of CHK1 in the replication stress response (80). In keeping with these data, MK-8776 in combination with cytarabine, led to reduced proliferation and impaired replication fork progression in AML blasts (75). It is noteworthy that MK-8776 also potentiated the effects of HDAC inhibitors in AML cell lines (81). The observation that granulocyte colony stimulating factor (GCSF) could force quiescent leukemic cells into the cell cycle and sensitize them to a CHK1 inhibitor (GDC-0575; Genentech)/cytarabine combination adds another interesting possibility for clinical evaluation (82).

#### CHK1/CHK2 inhibition in acute lymphoblastic leukemia

While CHK1 inhibitors have a wide cytotoxicity profile, leukemia cell lines were shown to be more sensitive to selective CHK1 inhibition (V158411 [Vernalis]) than lung and colon cancer cell lines (83). CHK1 is overexpressed and constitutively active in ALL cell lines and patient samples (73, 74). Inhibition of CHK1 with PF-0477736 (Pfizer) in this context resulted in reduced viability in cancer cell lines, but not in normal cells (73, 74). These results were corroborated in a xenograft mouse model (73) and an allograft mouse model (74) suggesting potential for clinical translation if a dosing schedule with minimal toxicity can be established. The CHK1 inhibitor prexasertib (Eli Lilly) exhibited single agent activity against both ALL cell lines and primary leukemic blasts, but not in healthy patient mononuclear cells (84). In cell lines prexasertib was synergistic with the tyrosine kinase inhibitor, imatinib, and purine nucleoside antimetabolite, clofarabine, with the prexasertib/imatinib combination also showing activity against primary leukemic blasts (84). Prexasertib, also acted as chemosensitizer in combination with doxorubicin in ALL cell lines and primary ALL cells (46). The combination of CHK1 inhibition with other targeted agents as well as immunotherapies against ALL are likely to be more promising than their combination with cytotoxic agents given the hematologic toxicity and the chemo resistant nature of the relapsed ALL.

#### CHK1/CHK2 inhibition in multiple myeloma

Targeting of CHK1 and CHK2 is also being actively studied in MM. AZD7762 was investigated in MM cell lines (including those with *TP53* loss) in combination with melphalan, bendamustine and doxorubicin (85). Combination with the CHK1 inhibitor potentiated the efficacy of all three cytotoxic agents confirming the hypothesis that MM cells rely on CHK1 to overcome DNA damage. It is noteworthy that AZD7762 did not synergize with bortezomib in this study. Given that PARP inhibitors *do* synergize with proteasome inhibitors in MM, this contrast highlights the need for clinical trials of DDR inhibitor combinations in MM to be guided by pre-clinical studies (85). Dai et al. proposed that inhibition of src could potentiate the activity of CHK1 inhibitors in MM, based on the observation that Chk1 inhibition activates the Ras/MEK/ERK pathway in this disease (86). Supporting this hypothesis, treatment with the src inhibitor dasatinib augmented sensitivity to CHK1 inhibition (with UCN-01) in MM cell lines, primary CD138+ cells from MM patients, and mouse xenograft mouse models (86).

#### CHK1/CHK2 inhibition in mature lymphoid neoplasms

Lymphoma cell lines, like leukemia cell lines, were shown to be more sensitive to selective CHK1 inhibition (with V158411) than lung and colon cell lines (83). MK-8776 enhanced the cytotoxicity of a variety of nucleoside analogs (fludarabine, cytarabine, gemcitabine) against CLL cell lines, including those with *TP53* mutations (87). More recently, MU380, an analogue of MK-8776, had potent single agent activity against *TP53* deficient CLL cells (88). These studies indicate the significant potential of CHK1 inhibition against high risk CLL, which remains an unmet clinical need.

AZD7762 treatment induces rapid DNA damage accumulation and apoptosis in DLBCL cell lines as well as primary samples (89). AZD7762 treatment also leads to apoptosis in MYC-deregulated mouse B-cell lymphoma cells *in vitro* and mitigates disease progression of p53 knockout, MYC deregulated B cell lymphoma transplant mouse models *in vivo (* 90). There was also a striking synergy with the PARP inhibitor, veliparib (AbbVie), in this setting (90).The role of CHK1 in the induction of HR following DSB (91) may be a plausible mechanism for the synergy of AZD7762 and veliparib but further studies would be required to verify this.

The dual CHK1/CHK2 inhibitor, PF-0477736 (Pfizer), is active in DLBCL cell lines and primary patient samples and also sensitizes *TP53* mutant DLBCL cell lines to doxorubicin (89). Notably, transfection of *TP53* wild-type cell lines with dominant-negative p53 did not result in increased sensitivity to PF-0477736 alone or with doxorubicin suggesting that this sensitivity is dependent on p53 loss, rather than p53 inactivation alone (89). CHK2 has also been noted to demonstrate cross-talk with ERK1/2 in DLBCL, with synergistic cytotoxicity of combined ERK and CHK2 inhibition in DLBCL cell lines, primary samples and *in vivo* models suggesting strong potential for clinical translation (92). In the EµMYC murine lymphoma model, disruption to the in the NF-κB pathway was correlated with resistance to CHK1 inhibition (93, 94). While these findings warrant validation in primary DLBCL specimens, they provide promise of generating robust biomarkers for use in clinical trials.

In MCL, PF-0477736 showed synergy with ibrutinib in sensitive cell lines, but only achieved cytostatic responses in ibrutinib resistant cell lines (95). A CHK1i-resistant MCL cell line (JEKO-1R), developed to be resistant to both PF-0477736 and AZD7762 (96), had an increased expression of genes involving pro-survival pathways and reduced cyclin D1 expression. Partial restoration of sensitivity to CHK1 inhibition by dasatinib treatment suggests that simultaneous targeting of other oncogenic signaling is worthy of exploration in combination with CHK1 inhibitors (96). Given the overlapping toxicity of CHK1 inhibitors and chemotherapy, combination strategies with other targeted agents could represent a promising opportunity.

### Targeting WEE1

WEE1 is a substrate in the DDR response cascade downstream of CHK1 (97). Its primary role is to prevent mitotic entry upon DDR by phosphorylating CDK1 to inhibit its function, and ultimately causing cell cycle arrest in G2 (97). WEE1 also plays an important role in the replication stress response, functioning similarly to CHK1 in maintaining genomic stability (1). Elevated expression of WEE1 has been observed in primary ALL patient samples and cell lines compared to normal mononuclear cells and bone marrow cells (98, 99), but otherwise this signaling axis is ostensibly functional across hematological malignancies. WEE1 inhibitors have reached clinical trials, though at least one has been terminated due to safety concerns (see **Table 1**). Several WEE1 inhibitors have been tested preclinically in hematological malignancies, and the results are discussed below.

**Table 1:** List of ongoing or completed clinical trials of WEE1 inhibitors in advanced hematological malignancies.

Pathway	Compounds	Latest Stage of Development and Trial Details	Clinical Trial Identifier(s)	Current Status (Ongoing or Published include reference if published)
Targeting WEE 1	AZD1775	Phase II as monotherapy or in combination with cytarabine in patients with AML and MDS	NCT02666950	Completed(not published)
	AZD1775	Phase II as monotherapy or in combination with Ara-C (cytarabine) in advanced AML, MDS and myelofibrosis	NCT03718143	Terminated (Safety concerns) Not published

The compounds targeting the checkpoint inhibitor pathway and its latest stage of development along with the trial details are indicated. AML, Acute Myeloid Leukemia; MDS, Myelodysplastic syndrome.

#### WEE1 inhibition in myeloid malignancies

While WEE1 inhibition as monotherapy appears to only have modest effects in AML cell lines, the WEE1 inhibitor AZD1775 (AstraZeneca) shows evidence of synergy with the PARP inhibitor olaparib (AstraZeneca) in primary AML samples as well as murine models (100), suggesting possibilities for DDRi combination trials in AML. Along these lines, siRNA knockdown of CHK1 and ATR enhanced the activity of AZD1775 against AML cells *in vitro (* 101). Similarly, combined treatment with inhibitors of CHK1 (MK-8776) and WEE1 (AZD1775) was synergistic against AML cells, likely through abrogation of the replication stress response. This data provides a potential rationale for evaluation of this combination in clinical trials, although overlapping toxicity will be a concern. AZD1775 has been shown to synergize with HDAC inhibitors (panobinostat, vorinostat and SBHA) against AML cells, independent of p53 status (102, 103). Importantly, this combination was shown to have no effect on normal CD34+ progenitors (102), and may thus have a favorable toxicity profile compared to the WEE1-CHK1 inhibitor combination.

#### WEE1 inhibition in acute lymphoblastic leukemia

WEE1 inhibition through AZD1775 induces apoptosis as a single agent in primary samples of B and T- ALL (98). As with AML, described above, combinations of WEE1 inhibitors with other DDR inhibitors appear active in ALL. A combination of olaparib and AZD1775 was synergistic against ALL cells *in vitro* (100) and a combination of the CHK1 inhibitor PF-0477736 with AZD1775 showed synergy against primary ALL samples (104). WEE1 inhibition also induced changes in the metabolism of T-ALL cells resulting in increased dependence on glutaminolysis. Dual inhibition of WEE1(with AZD1775) and glutaminase (with BPTES) showed synergy in T-ALL cell lines, and in patient-derived xenograft mouse models, supporting this hypothesis (99). Further studies exploring alterations in cellular metabolism during DDR inhibition are warranted.

For immediate clinical development, combinations of WEE1 inhibition with chemotherapeutics are promising. AZD1775 synergized with cytarabine, among other cytotoxic agents, against T-ALL cell lines and cell line xenograft models (105). In ALL cell lines, AZD1775 synergized with doxorubicin to induce apoptosis associated with increased mitotic entry and deregulation of the NOTCH pathway, with minimal effects on normal progenitors (106).These data support the combination of WEE1 inhibitors with conventional chemotherapeutics for evaluation in clinical trials of relapsed or refractory ALL.

#### WEE1 inhibition in multiple myeloma

AZD1775 has single agent activity against bortezomib/lenalidomide resistant MM cell lines and patient samples, inducing apoptosis regardless of p53 status (107). AZD1775 also sensitized MM cells to bortezomib and synergized with the HDAC inhibitor, vorinostat, indicating potential strategies for combination trials (108). Separately, WEE1 inhibition may also be a combination partner for antibody-drug conjugates. MEDI2228 (described previously in the ATM section) induces significant levels of DNA damage with DDR pathway activation and reduces the viability of both cell lines and patient samples (32). Inhibition of WEE1 was shown to synergize with MEDI2228, suggesting potential for clinical combination therapy (32). Given its role in the response to replication stress, WEE1 inhibitors may have greater efficacy in those subsets of MM with greater replication stress. Further pre-clinical studies would be required before these agents can progress to clinical trials in MM.

#### WEE1 inhibition in mature lymphoid neoplasms

In the setting of MCL, the combination of AZD1775 and the CHK1 inhibitor PF-0477736 was synergistic *in vitro* and in PDX models (109). In DLBCL, the same combination was not only shown to induce apoptosis but also to destabilize MYC protein (110). Interestingly the sensitivity to the combination did not correlate with the degree of MYC expression or molecular subtype suggesting that factors other than MYC-induced replicative stress may be involved. AZD1775 increased the dependency of DLBCL cells on BCL-2 and MCL-1, with enhanced apoptosis when AZD1775 was combined with BCL2(venetoclax/navitoclax) or MCL-1 (S63845) inhibitors (111). AZD1775 also synergized with CHOP (doxorubicin in particular), radiotherapy or rituximab in DLBCL cell lines (112). These data provide a framework for the design of combination clinical trials with WEE1 inhibitors in lymphoma, although further pre-clinical studies are needed to identify the appropriate molecular subtypes of DLBCL for a particular WEE1-based combination.

### Targeting Poly-ADP-ribose polymerase (PARP)

PARP is a DDR effector protein that interacts directly with damaged DNA and catalyses the formation of poly(ADP-ribose) chains on itself and other proximal proteins; this post-translation modification functions to recruit other DDR-related proteins (6). Inhibition of PARP has gained prominence in the field of solid tumors through its effectiveness, particularly against BRCA-deficient cancers (6). Though BRCA deficiency is not a common feature in hematological malignancies (113), the expression of PARP is found to be aberrant in blood cancers. It is overexpressed in AML cell lines and patient samples (114, 115), and this expression can be augmented by oncogenic KRAS (116). Increased PARP expression has also been observed in CML cell lines (117). Importantly, high PARP levels have been associated with poor clinical outcome in AML (114) and a poor early response to treatment in pediatric ALL (118). Considering these observations, it is not surprising that PARP inhibitors have been evaluated extensively in several hematological malignancies, as described below.

#### PARP inhibitors and myeloid malignancies

The PARP inhibitor olaparib demonstrated efficacy against AML cell lines as well as primary samples with high phospho- gamma H2AX levels (115). Mutational and gene expression profiling has identified a BRCA-deficient subset of AML which may be more sensitive to PARP inhibition (119). Consistent with this, BCAT1 overexpression resulted in a BRCA deficient phenotype and was associated with increased sensitivity of AML cell lines to talazoparib (Pfizer) as a single agent as well as in combination with daunorubicin (120). Similarly, another PARP inhibitor, KU-0058948, had efficacy against both AML cell lines and primary samples due to defects in the HR pathway, with synergism noted along with the HDAC inhibitor, MS275 (121).

Interestingly, in the context of transformed primary mouse hematopoietic cells, specific translocations appear to affect dependency on PARP activity. The presence of either AML1-ETO and PML-RARα fusion genes were associated with sensitivity to PARP inhibitors (olaparib and veliparib) whereas AML driven by MLL fusions were resistant to PARP inhibition (122). The KM2A-AF9 fusion protein resulting from the MLL rearrangement was shown to upregulate HOXA9, a transcription factor known to upregulate the HR recombinase, RAD51, and thus possibly confer resistance to PARP inhibition (122, 123). These studies highlight the variability of response to PARP inhibitors *in vitro* and suggest that biomarker driven clinical trials may be a valid strategy for clinical translation of PARP inhibition for AML.

Overall, the pre-clinical efficacy of PARP inhibitors is lower in myeloproliferative neoplasms (MPN) when compared to AML. BCR-ABL driven leukemias (e.g. CML) are known to have upregulation of alt-NHEJ related proteins including DNA Ligase IIIα and PARP (117). CML primary samples and cell lines required simultaneous inhibition of PARP (with NU1025) and DNA ligase IIIα (with L67) to reduce survival (117). The efficacy of these inhibitors was correlated with the expression of these proteins. Talazoparib, and olaparib also had modest activity against cell lines and primary samples of Philadelphia negative MPN (124). One study demonstrated that almost 50% of MPN cases had impaired formation of RAD51 foci in response to ionizing radiation, which was associated with sensitivity to olaparib and veliparib (125). Clinical trials evaluating the efficacy of PARP inhibitors in CML should therefore be stratified by such biomarkers and could potentially be conducted in combination with BCR ABL inhibitors.

#### PARP inhibition in acute lymphoblastic leukemias

The translocation t (17,19) (q22;p13) results in the formation of the TCF3-HLF fusion protein and characterizes a rare subtype of pediatric ALL (< 1%) (126). TCF3-HLF expression impairs HR repair (127). Congruent with this, olaparib and veliparib showed significant activity against TCF3-HLF B-ALL *in vitro* (127), and in combination with temozolomide in TCF3-HLF xenograft models (127). The combination of the PARP inhibitor, rucaparib (Clovis), with 5-fluorouracil showed significant activity against T-ALL cell lines and primary allograft and xenograft animal models, with preferential effects on leukemic blasts in comparison to normal mononuclear cells (128). 5 Fluorouracil is however not a standard treatment for T-ALL; hence the clinical applicability of this regimen is doubtful. Evaluation of PARP inhibitors with agents in clinical use for relapsed T-ALL is hence required.

LIM domain only 2 (LMO2) is a cysteine rich protein implicated in the maintenance of hematopoietic stem cells (129, 130). LMO2 is deregulated in T-ALL associated with translocations involving chromosome 11p and in B-ALL with t (17,19) (131, 132). LMO2 inhibits recruitment of BRCA1 to sites of DSB via an interaction with 53BP1 (130). The HR deficiency occurring as a result of LMO2 expression rendered T-ALL cells more sensitive to PARP inhibition (using olaparib), both as a single agent as well as in combination with doxorubicin (130). However in another study, PARP inhibition (with PJ-34) did not induce apoptosis in unselected B and T- ALL cell lines as single agents or in combination with a NOTCH inhibitor (DAPT) (133). These data suggest that PARP inhibition may represent an option for clinical trials focusing on ALL with LMO2 overexpression.

#### PARP inhibition in multiple myeloma

Single-agent olaparib induced DSBs and apoptosis at low micromolar concentrations in both MM cell lines and primary samples, particularly in the setting of MYC overexpression (134). Genome wide loss of heterozygosity (LOH) is associated with dysfunctional HR and sensitivity to PARP inhibitors; LOH occurs in MM and is indeed associated with dysfunctional HR in a subset of patients (135). This lends credence to the rationale for clinical evaluation of PARP inhibitors in this disease. However, the incorporation of PARP inhibitors into treatment protocols will likely need to be done in combination with currently used agents for MM, and there is pre-clinical evidence for the efficacy of PARP inhibitor combinations.

The combination of bortezomib with veliparib or olaparib was associated with increased cytotoxicity (compared to either drug alone) in both MM cell lines and cell line xenografts (136–138). The underlying mechanism is postulated to be that proteosome inhibition induces impairment of HR in MM cells through abrogation of γ-H2AX polyubiquitylation (136, 139). PARP inhibitors (PJ-34, bufalin) have also been combined with melphalan and other cytotoxic agents showing synergy in multi-drug resistant MM cell lines (140–142). Additional targeting of NHEJ using a DNA–PK inhibitor, NU7026, augmented the cytotoxicity of the melphalan/veliparib combination in drug resistant MM cell lines and cell line xenografts (143), suggesting that dual DDR inhibitor combinations may also be attractive options for evaluation in clinical trials. Separately, the CDK inhibitor, dinaciclib, was shown to impair the expression of HR related genes and sensitize MM cells to veliparib *in vitro* and *in vivo* (144). As CDK inhibitors such as dinaciclib have single agent efficacy in MM (144, 145) and are being studied actively as a therapeutic option, further mechanistic and pre-clinical studies of PARP inhibitors combined with other CDK inhibitors are warranted.

#### PARP inhibition in mature lymphoproliferative neoplasms

ATM deficient tumors are also hypothesized to be susceptible to PARP inhibition (1, 146). ATM deficient CLL cells showed increased *in vitro* sensitivity to olaparib compared to those with intact ATM (147). These findings have also been confirmed in murine models of ATM deficient CLL (148). Interestingly, although CLL cells were also sensitive to talazoparib, the correlation of cytotoxicity with ATM loss was less clear in this setting (149). These data suggest that synthetic lethality profiles may differ between PARP inhibitors, and that clinical responses to such in lymphoid malignancies will be heterogeneous. Careful patient selection based on robust biomarkers will be required for the optimal design of clinical trials.

In keeping with the CLL, ATM loss in MCL was shown to be associated with *in vitro* sensitivity to PARP inhibitors (PJ-34, olaparib) (150). Interestingly, PARP inhibition with olaparib was shown to be even more effective in MCL cell lines with combined loss of ATM and *TP53* than those with ATM loss alone (151). As loss of *TP53* is known to herald a poor prognosis in MCL (152), and since olaparib synergized with the BTK inhibitor, ibrutinib, in MCL cell lines (153), these data provide promising grounds for the design of a PARP inhibitor-BTK combination in this high risk subset of MCL.

In the setting of unselected DLBCL cell lines, olaparib showed limited single agent activity but potential for cell kill when combined with rituximab (154). As with T-ALL (described above), LMO2 expression impaired HR in DLBCL and conferred sensitivity to PARP inhibition (with olaparib) as a single agent as well as in combination with genotoxic agents (doxorubicin alone or the R-CHOP regimen) (130). As LMO2 expression is an independent poor prognostic factor in DLBCL treated with the R-CHOP regimen (155), there is a rationale for the evaluation of PARP inhibition in combination with chemo-immunotherapy in LMO2-expressing DLBCL. Gemcitabine, melphalan and busulphan (Gem/Mel/Bu) is a potent conditioning regimen for relapsed lymphomas (156), and addition of olaparib to Gem/Mel/Bu resulted in a three-fold reduction in lymphoma cell line proliferation *in vitro (* 157). Although these data are promising, the potential hematologic toxicity of this regimen would be a concern when considering clinical evaluation.

### Clinical trials investigating DDR inhibitors in hematologic malignancies

Numerous clinical trials are currently in progress evaluating DDR inhibitors in hematologic malignancies. Trials involving PARP inhibitors either as single agents or in combination comprise the majority, with ATR inhibitors also being actively investigated. These clinical trials are summarized in **Tables 1**–**5**, and key findings from the reported trial results as well as major points of interest regarding the ongoing trials are discussed below.

**Table 2:** List of ongoing or completed clinical trials of ATR inhibitors in advanced hematological malignancies.

Pathway	Compounds	Latest Stage of Development and Trial Details	Clinical Trial Identifier(s)	Current Status (Ongoing or Published include reference if published)
HRR (Targeting ATR)	BAY1895344	Phase I as monotherapy in patients with advanced solid tumors and lymphomas	NCT03188965	Recruiting
	AZD6738	Phase I as monotherapy in patients with R/R CLL, Prolymphocytic leukemia (PLL) or B cell lymphomas	NCT01955668	Completed(not published).
	AZD6738	Phase I/II as monotherapy or in combination with Acalabrutinib in R/R high-risk CLL	NCT03328273	Active, not recruiting
	AZD6738	Phase I as monotherapy in the treatment of MDS or Chronic Myelomonocytic Leukemia (CMML)	NCT03770429	Recruiting
	AZD6738	Phase I as combination therapy with Paclitaxel in refractory cancer	NCT02630199	Recruiting

The compounds targeting the HRR pathway and its latest stage of development along with the trial details are indicated. ATR, Ataxia Telangiectasia and Rad3-related; CLL, Chronic Lymphocytic Leukemia; MDS, Myelodysplastic syndrome; R/R, Relapsed or Refractory.

**Table 3:** List of ongoing or completed clinical trials of DNA-PK inhibitors in advanced hematological malignancies.

Pathway	Compounds	Latest Stage of Development and Trial Details	Clinical Trial Identifier(s)	Current Status (Ongoing or Published include reference if published)
NHEJ (Targeting DNA-PK)	MSC2490484A	Phase I as monotherapy in advanced solid tumors or CLL	NCT02316197	Completed (158)
NHEJ (Targeting DNA-PK and mTOR kinase)	CC-115	Phase I as monotherapy in advanced solid tumors or CLL	NCT01353625	Completed

The compounds targeting the various pathways and its latest stage of development along with the trial details are indicated. CLL, Chronic Lymphocytic Leukemia; DNA-PK, DNA-dependent protein kinase; NHEJ, Non-homologous end joining; mTOR, mammalian target of rapamycin.

**Table 4:** List of completed clinical trials of CHK1 inhibitors in advanced hematological malignancies.

Pathway	Compounds	Latest Stage of Development and Trial Details	Clinical Trial Identifier(s)	Current Status (Ongoing or Published include reference if published)
Targeting CHK1	MK-8776	Phase II as cytarabine monotherapy or in combination with MK-8776 in adult patients with relapsed AML	NCT01870596	Completed (159)
	SRA737	Phase II for Advanced Solid Tumors or NHL	NCT02797964	Completed(not published)

The compounds targeting the checkpoint inhibitor pathway and its latest stage of development along with the trial details are indicated. CHK1, Checkpoint kinase 1; AML, Acute Myeloid Leukemia; NHL, Non-Hodgkin Lymphoma.

**Table 5:** List of ongoing or completed clinical trials of PARP inhibitors in advanced hematological malignancies.

Pathway	Compounds	Latest Stage of Development and Trial Details	Clinical Trial Identifier(s)	Current Status (Ongoing or Published include reference if published)
BER (Targeting PARP).	talazoparib	Phase I in patients with advanced hematological malignancies (AML, MDS, CLL, MCL)	NCT01399840	Completed (160)
	talazoparib	Phase I in patients with AML or MDS that have a mutation in the cohesin complex	NCT03974217	Ongoing
	talazoparib	Phase I/II in combination with Decitabine in patients with untreated and refractory/relapsed (R/R) AML	NCT02878785	Ongoing
	talazoparib	Phase I/Ib in combination with gemtuzumab ozogamicin in patients with R/R CD33 positive AML	NCT04207190	Active, recruiting
	veliparib	Phase I in combination with Temozolomide in patients with acute leukemia	NCT01139970	Active, not recruiting
	veliparib	Phase I in combination with Bortezomib (proteasome inhibitor) in multiple myeloma	NCT01495351	Completed (161)
	veliparib	Phase I as monotherapy in refractory solid tumors or hematologic cancers	NCT00387608	Completed (162)
	veliparib	Phase I in combination with Cyclophosphamide in refractory solid tumors and lymphoid cancers (lymphoma and CLL)	NCT01445522	Completed (not yet published)
	veliparib	Phase I in combination with Topotecan in refractory solid tumors, lymphomas and CLL	NCT00553189	Completed (163)
	veliparib	Phase I in combination with Topotecan +/- Carboplatin in R/R acute leukemia, high-risk myelodysplasia, or aggressive myeloproliferative disorders	NCT00588991	Active, not recruiting
	veliparib	Phase I/II in combination with Bendamustine Hydrochloride +/- Rituximab in lymphoma, multiple myeloma or R/R solid tumors	NCT01326702	Completed (164)
	veliparib	Phase I in combination with Cyclophosphamide and Doxorubicin in metastatic/unresectable solid tumors or NHL	NCT00740805	Active, not recruiting.
	veliparib	Phase II in combination with Topotecan, Carboplatin +/- Veliparib in advanced myeloproliferative disorders, AML or chronic myelomonocytic leukemia	NCT03289910	Active, not recruiting
	veliparib	Phase I/Ib in combination with Nivolumab in patients with advanced refractory solid cancers and lymphoma	NCT03061188	Active, not recruiting
	CEP-9722	Phase I in combination with Gemcitabine and Cisplatin in advanced solid tumors or MCL	NCT01345357	Completed(not published)
	olaparib	Phase II as monotherapy in Isocitrate Dehydrogenase (IDH) mutant relapsed or refractory AML and MDS	NCT03953898	Recruiting
	olaparib	Phase II as monotherapy in solid tumors, NHL or histiocytic disorders with defects in DDR genes	NCT03233204	Recruiting
	olaparib	Phase I in combination with high dose chemotherapy (Olaparib, Vorinostat, Gemcitabine, Busulfan and Melphalan) in R/R lymphomas undergoing stem cell transplant	NCT03259503	Ongoing

The compounds targeting the BER pathway and its latest stage of development along with the trial details are indicated. ALL, Acute Lymphoblastic Leukemia; AML, Acute Myeloid Leukemia; BER, Base excision repair; CLL, Chronic Lymphocytic Leukemia; DDR, DNA Damage Repair; MCL, Mantle Cell Lymphoma; MDS, Myelodysplastic syndrome; NHL, Non-Hodgkin Lymphoma; PARP, poly ADP ribose polymerase; R/R, Relapsed or Refractory.

PARP inhibitors are the most advanced among DDR inhibitors in terms of clinical development in hematological cancers (165). Following a successful phase 0 trial demonstrating PARP inhibition histologically in tumor biopsies (162), a number of phase 1 studies have sought to evaluate the safety and tolerability of veliparib in advanced cancer. Myelosuppression has been the key dose limiting toxicity (DLT) identified in these studies with some efficacy signals also seen (163). Among two patients with lymphoid malignancies included in a phase 1 trial of veliparib with oral cyclophosphamide, one patient with CLL/SLL achieved prolonged stable disease (166).

Veliparib (V) was also combined with bendamustine (B) +/- rituximab (R) in relapsed/refractory patients with solid malignancies, lymphoma or MM (164). There were 14 lymphoma patients and 1 MM patient among the 34 recruited, 5 out of 7 patients on the VB arm achieved an objective response while 6 out of 7 patients on the VBR arm achieved an objective response. The single MM patient achieved a partial response (PR). The combination was well tolerated with grade >3 neutropenia and thrombocytopenia occurring in 12.2% and 9.8% of patients respectively. Given that BR is an established treatment regimen in indolent B-cell lymphomas, this combination would be a promising option to be taken forward into phase II trials.

Based on pre-clinical data showing synergy between bortezomib and PARP inhibitors, Neri et al. conducted a phase 1 trial evaluating veliparib in combination with bortezomib/dexamethasone in relapsed or refractory MM (161). The combination was well tolerated and the overall response rate of 39% was encouraging given that close to 80% of the patients had prior bortezomib treatment. Importantly, patients with mutations in HR related genes were found to have a higher response rate, supporting the biological rationale for the study (161). Future phase II studies using this combination should ideally employ similar biomarkers to better select patients.

Veliparib is also being studied in combination with topotecan/carboplatin in MPN, AML and chronic myelomonocytic leukaemia in two phase II trials (NCT03289910 and NCT03289910). These ongoing trials will provide valuable data on the potential synergy between veliparib and DNA damaging chemotherapy. Haematologic toxicity may however be a concern with these combinations.

Olaparib was evaluated in a phase 1 trial in patients with relapsed CLL (n=9), T-PLL (n=2) or MCL (n=4) (167). The treatment was well tolerated with grade 3 hematologic toxicities occurring in ten patients. In patients with the ATM or SF3B1 mutations, a longer median survival time of 192 days was seen compared to 89 days in the unmutated group. These data suggest ATM and SF3B1 mutations may be a potential biomarker for patient selection in future studies of PARP inhibitors in lymphoma. It is noteworthy that Olaparib is being studied in an ongoing phase I trial (NCT03259503) in combination with vorinostat, busulphan, melphalan and gemcitabine in patients with relapsed lymphoma undergoing stem cell transplant. While the synergy between Olaparib and the histone deacetylase (HDAC) inhibitor would be of interest, haematologic toxicity is certainly a concern. Olaparib is also under investigation as monotherapy in an ongoing phase II trial (NCT03953898) for patients with relapsed AML with isocitrate dehydrogenase mutations. If this trial shows promising results, the combination of Olaparib with IDH inhibitors maybe a consideration for future studies. Talozaparib monotherapy was studied in a phase 1 trial involving patients with AML, MDS, CLL and MCL (160). Hematologic toxicities were again the key DLTs, and stable disease was seen in patients with AML, MDS and MCL while transfusion independence was reported in patients with AML and MDS. Taken together, these data suggest that PARP inhibitors have limited clinical efficacy as monotherapy or in combination with cytotoxic agents in hematologic malignancies.

Combining PARP inhibitors with other targeted agents in acute leukemias is therefore a strategy being pursued. Talozaparib is being studied in a phase I/II trial in combination with decitabine (NCT02878785) in relapsed AML as well as with gemtuzumab ozogomycin (NCT04207190), in relapsed CD33 positive AML. These trials are currently ongoing, and their results will demonstrate the potential synergy between PARP inhibitors and other targeted therapies for AML. The combination with gemtuzumab in particular would be of interest for the core binding factor acute leukaemias given their success in the frontline setting (168).Combinations of PARP inhibitors with immunotherapy are particularly worthy of exploration given that hematologic toxicities with the latter are not as prominent as with cytotoxic chemotherapy.

Beyond PARP inhibitors, the majority of the reported clinical data are for the CHK1 and DNA-PK inhibitors. Based on the pre-clinical data previously discussed (80), the combination of the CHK1 inhibitor MK-8776 with cytarabine was evaluated in a phase 1 trial in relapsed or refractory AML. Promising safety and preliminary efficacy results were seen in this study, prompting the advancement of this combination to the phase II setting (169). In a randomized phase II comparison of cytarabine as a single agent with cytarabine + MK-8776, Webster et al. demonstrated a significant increase in DNA damage in the combination arm based on γH2AX expression (159). Interestingly, no significant difference in complete response rates were seen between the arms: (36% and 44% for the cytarabine and combination arms respectively). Definitive conclusions on therapeutic benefit may be difficult to draw based on the small number of patients enrolled in the study (n=32), hence larger randomized studies are called for to further evaluate this promising combination.

Several trials are evaluating ATR inhibitors as monotherapy and in combination with cytotoxic or targeted agents. The combination with acalabrutinib in relapsed high risk CLL (NCT03328273) is of particular interest as these agents are less likely to have overlapping toxicities and may have potential synergy, especially in the more aggressive subsets with high levels of replication stress.

Based on promising preclinical data, the DNA-PK/MTORK inhibitor CC-115 was studied in a phase 1 trial which included eight patients with ATM mutant CLL (68, 170). Two patients achieved a partial remission (PR) while three achieved a PR with lymphocytosis. Although preliminary, these results in a heavily pretreated population of high risk CLL suggest this agent is worthy of further evaluation in phase II studies.

### Conclusions & future directions

In conclusion, we present an overview of promising pre-clinical and early phase clinical trial data supporting the use of DDR inhibitors as a viable treatment modality in haematological malignancies. We consider the following as priorities for evaluation in future clinical trials. Targeting replication stress via ATR,CHK1 or WEE1 inhibition would be of value in MM, given the importance of replication stress in this malignancy. CHK1/WEE1 inhibitors also show promise in B-cell lymphomas and would be of particular interest in MYC driven subsets given their high replication stress. The combination of these agents with immunotherapy in the B-cell malignancies would also be worthy of exploration. Combining ATR inhibitors with inducers of replication stress would be of interest in AML, given the *in-vitro* synergy that has been reported, especially in elderly patients who are not suitable for intensive chemotherapy. Targeting ATM maybe synthetically lethal with MLL rearrangements in acute leukaemias, and this strategy should be studied further in this subgroup of patients.

Identifying the right patient population, disease subsets, and combinatorial drug partners are key questions that need to be answered before these agents can move into mainstream clinical practice. The validation of robust biomarkers for sensitivity to DDR inhibitors will be a key step forward and should be used in the design of future clinical trials (171).

A better understanding of the cross talk between DDR pathways in specific tumors as well as how this manifests in terms of mutational, gene expression or proteomic profiling will also be critical for this purpose (172). Ex vivo drug sensitivity testing in association with artificial intelligence based predictive models provides an exciting opportunity for personalized cancer therapeutics (173–175). Such platforms create a vast drug combination search space and can discover efficacious combinations agnostic of underlying mechanisms. Evaluation of DDR inhibitors using such platforms is therefore a promising arena for future research and has the potential to identify new DDRi-DDRi, DDRi—chemotherapy drug combinations. In addition to personalized recommendations, recurring combinations identified can also be used to uncover novel biomarkers of sensitivity that may be applicable to the wider population of cancer patients.

Challenges to be overcome with such ex vivo approaches include recreating the immune microenvironment, which plays a crucial role in drug sensitivity in hematologic malignancies (176) hence the development of such platform enhancements are a pressing need. In support of this, there is growing interest in the interaction between the DDR and immune dysregulation, providing opportunities for simultaneous targeting of immune checkpoints with DDR inhibition (177). A better understanding of how deregulation of DDR pathways affects immune checkpoints in specific malignancies will be central to taking this field forward.

## Author contributions

SD: Conceptualization, Writing – original draft. AL: Writing – original draft. JT: Writing – original draft. RT: Writing – original draft. LP: Writing – review & editing. EC: Writing – review & editing. JL: Writing – review & editing. YC: Writing – review & editing. SL: Writing – review & editing. PJ: Writing – original draft. AJ: Conceptualization, Writing – original draft, Writing – review & editing.
